# Economic evaluation of toripalimab combined with chemotherapy in the treatment of non-small cell lung cancer

**DOI:** 10.3389/fpubh.2023.1137255

**Published:** 2023-03-24

**Authors:** Hao Wang, Yunchun Long, Yuan Xu, Li Liao, Yujie Zhou

**Affiliations:** ^1^Department of Pharmacy, China Pharmaceutical University Nanjing Drum Tower Hospital, Nanjing, Jiangsu Province, China; ^2^Department of Pharmacy, Nanjing Drum Tower Hospital, Nanjing, Jiangsu Province, China; ^3^Department of Respiratory and Critical Care Medicine, Nanjing Drum Tower Hospital, Nanjing, Jiangsu Province, China

**Keywords:** cost-effectiveness analysis, toripalimab, partition survival model, non-small cell lung cancer, chemotherapy

## Abstract

**Background and objective:**

The CHOICE-01 trial showed that toripalimab plus chemotherapy achieved satisfactory outcomes compared with chemotherapy in patients with advanced non-small cell lung cancer (NSCLC) who were negative for driver genes, but the economics of this regimen is unclear. Therefore, this study aimed to evaluate the cost-effectiveness of toripalimab in combination with chemotherapy in advanced NSCLC with negative driver genes from the perspective of the Chinese healthcare system.

**Materials and methods:**

A three-state partitioned survival model was developed to simulate the costs and outcomes associated with adding toripalimab to first-line chemotherapy. The clinical data in the model came from the CHOICE-01 trial, only direct medical costs were included, and utility values were referred to the literature. Four models were applied to explore the differences in the results of fitting and extrapolating K-M curves from different models, and cost-effectiveness subgroup analysis was performed. The incremental cost-effectiveness ratio (ICER) was used as the main outcome measure. Sensitivity analysis was performed to assess the impact of parameter uncertainty on the model.

**Results:**

The baseline analysis showed that toripalimab coupled with chemotherapy cost $21,052 more than chemotherapy ($43,197 vs. $22,145) and also gained 0.71 QALYs more (1.75 QALYs vs. 1.03 QALYs), with an ICER of $29,478/QALYs. At the current willingness-to-pay threshold ($35,108/QALY), the extra cost was well worth it. The results of fitting and extrapolating the survival curves using other models were consistent with the results of the standard parametric model. Subgroup analysis demonstrated that the addition of toripalimab to chemotherapy was economical. Sensitivity analysis showed that the utility values of PD and PFS stages had the greatest impact on the model.

**Conclusion:**

From the viewpoint of the Chinese healthcare system, toripalimab combined with chemotherapy in the treatment of advanced NSCLC with negative driver genes was likely to be cost-effective compared with chemotherapy.

## 1. Introduction

Lung cancer is a kind of malignant tumor with high morbidity and mortality worldwide. According to the data released by the International Agency for Research on Cancer of the World Health Organization in 2020, lung cancer ranks second in incidence and first in mortality among malignant tumors in the world (1). On the grounds of the “Cancer Incidence and Mortality in China 2016” released by the National Cancer Center of China in 2022, there are 828,000 new cases of lung cancer and 657,000 death cases each year in China, ranking first in both incidence and mortality of cancer (2). On the basis of the development trend in recent decades, the incidence and mortality of lung cancer in China have increased year by year (3, 4). In addition to high morbidity and mortality, the economic burden of lung cancer is extremely heavy. A study showed that the economic burden caused by lung cancer was as high as $25.069 billion in 2017, accounting for about 1.43% of China’s healthcare expenditure, and the total economic burden related to lung cancer is expected to increase to $40.4 billion and $531 billion by 2025 and 2030, respectively (5).

Non-small cell lung cancer (NSCLC), mainly including lung adenocarcinoma (LUAD), lung squamous cell carcinoma (LUSC), and large cell carcinoma, is the main subtype of lung cancer, accounting for about 80–85% of all cases (6, 7). Due to the atypical symptoms, most patients have missed the opportunity of surgical treatment at the time of diagnosis, and the 5-year survival rate is less than 20% (8, 9).

For decades, the standard first-line treatment for NSCLC has been platinum-based two-agent chemotherapy, but the objective response rate (ORR), progression-free survival (PFS), and overall survival (OS) have been disappointing (10). Fortunately, with the development of precision therapy and immunotherapy in recent years, the prognosis of patients with advanced NSCLC has been greatly improved. PD-(L)1 inhibitor is one of the most commonly used drugs in immunotherapy. Many clinical trials have shown that the addition of PD-(L)1 inhibitors to chemotherapy can not only prolong the OS and PFS of patients with NSCLC but also reduce the incidence of adverse reactions (11–14). Toripalimab, a domestic anti-PD-1 monoclonal antibody, was marketed in China in 2018 and included in China’s national insurance directory in 2020. The CHOICE-01 trial validated the efficacy and safety of toripalimab combined with chemotherapy in patients with advanced NSCLC with negative driver genes (15). Based on this trial, the National Medical Products Administration (NMPA) of China has approved toripalimab in combination with pemetrexed and platinum as the first-line treatment for patients with inoperable, locally advanced, or metastatic NSCLC that is negative for epidermal growth factor receptor (EGFR) mutations and anaplastic lymphoma kinase (ALK). Although toripalimab is currently the cheapest compared with other PD-(L)1 inhibitors, its economics are not clear. Therefore, this study aimed to evaluate the cost-effectiveness of toripalimab coupled with chemotherapy in the treatment of NSCLC from the perspective of the Chinese healthcare system.

## 2. Materials and methods

### 2.1. Model overview

**Figure 1:**
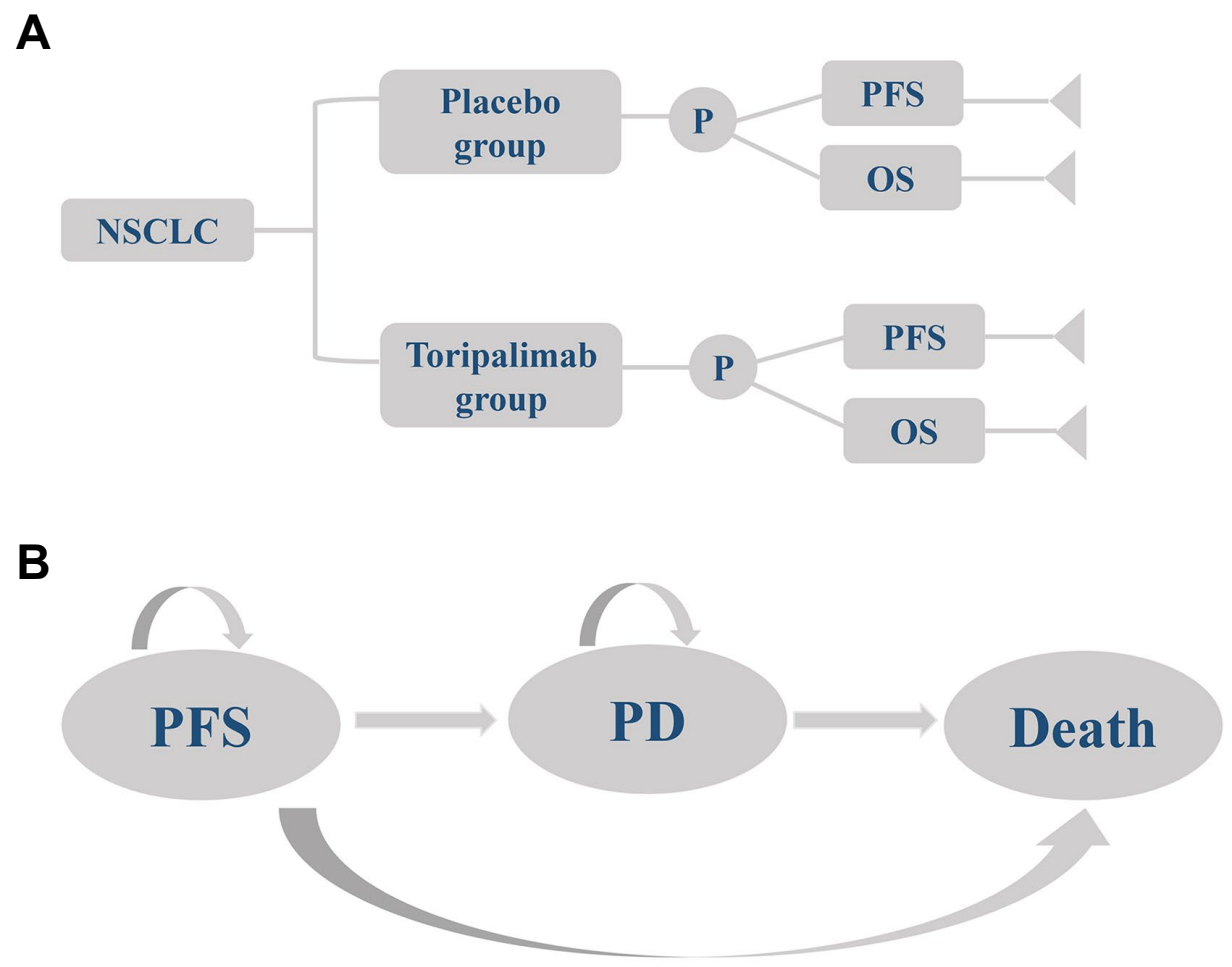
A three-state partitioned survival model simulating advanced non-small cell lung cancer. **(A)** Decision tree and **(B)** Graph of the partitioned survival model. NSCLC, non-small cell lung cancer; placebo group, placebo + chemotherapy; toripalimab group, toripalimab + chemotherapy; PFS, progression-free survival; PD, progressive disease; OS, overall survival.

A partitioned survival model including progression-free survival (PFS), progressive disease (PD), and death was established in the TreeAge Pro 2020 to simulate the cost-effectiveness of two first-line therapies for advanced NSCLC (Figure 1). The baseline characteristics of the patients in the model were consistent with those in the CHOICE-01 trial, and medication was administered as follows: (1) Toripalimab group: Squamous NSCLC patients received toripalimab (240 mg, every 3 weeks), nabpaclitaxel (100 mg/m^2^, days 1, 8, and 15, every 3 weeks) and carboplatin (AUC five, every 3 weeks) for 4 cycles, followed by maintenance of toripalimab every 3 weeks. Non-squamous NSCLC received toripalimab (240 mg, every 3 weeks), pemetrexed (500 mg/m^2^, every 3 weeks), and cisplatin (75 mg/m^2^, every 3 weeks) or carboplatin (AUC five, every 3 weeks) for 4 cycles, followed by maintenance of toripalimab plus pemetrexed every 3 weeks. (2) Placebo group: Squamous NSCLC patients received placebo (240 mg, every 3 weeks), nabpaclitaxel (100 mg/m^2^, days 1, 8, and 15, every 3 weeks), and carboplatin (AUC five, every 3 weeks) for 4 cycles, followed by maintenance of placebo every 3 weeks. Non-squamous NSCLC received placebo (240 mg, every 3 weeks), pemetrexed (500 mg/m^2^, every 3 weeks) and cisplatin (75 mg/m^2^, every 3 weeks) or carboplatin (AUC five, every 3 weeks) for 4 cycles, followed by maintenance of placebo plus pemetrexed every 3 weeks (15). All patients were assumed to enter the model in PFS status and receive two first-line therapies until disease progression or intolerable adverse events (AEs). Because of the complexity of treatment after disease progression and the presence of crossover but unclear crossover proportions, we made the following assumptions on the ground of the CHOICE-01 trial: (1) Toripalimab group: 1.6% of patients continued toripalimab, 33.7% received docetaxel (75 mg/m^2^, every 3 weeks), and 64.7% received best supportive care (BSC). (2) Placebo group: 52% of patients received toripalimab and 48% received docetaxel (75 mg/m^2^, every 3 weeks) (15). All drugs were given intravenously.

The model period was set as 3 weeks, in line with the CHOICE-01 trial, and the study time horizon was set as 10 years. With incremental cost-effectiveness ratio (ICER) as the main index, the meaning of the ICER for every increase a QALY gain need to pay how much money, calculation formula is ICER = (C1 − C2)/(E1 − E2). In pharmacoeconomic evaluation, it is often necessary to compare ICER with the willingness-to-pay (WTP) threshold. If ICER is less than the threshold, the economy of the intervention group is better than that of the control group. Other indicators included total cost, incremental cost, life years (LYs), quality-adjusted life years (QALYs), and incremental QALYs. The WTP was set to three times of China’s GDP *per capita* in 2021 (WTP = $35,108/QALY), and the cost and utility values were discounted at an annual discount rate of 5%, according to the recommendations of the World Health Organization (WHO) and China Guidelines for Pharmacoeconomic Evaluations (16).

### 2.2. Survival estimate

The data of effectiveness and safety in the model came from the CHOICE-01 trial (15). First, points were taken from the K-M curves in the CHOICE-01 trial using Engauge Digitizer software.^1^ Then the survHE package in the R software^2^ was called to reconstruct the individual patient data according to the survival rate, time, sample size, and number of people at risk. Finally, exponential distribution, Weibull distribution, lognormal distribution, log-logistic distribution, and Gompertz distribution fitted hazard functions to survival curves. See Supplementary Table 1 for Akaike Information Criterion (AIC) and Bayesian Information Criterion (BIC) for different distribution risk functions. According to AIC and BIC, combined with a visual inspection, log-logistic distribution was selected as the optimal fit of the PFS curve in the placebo group, and the lognormal distribution was chosen as the best fit for the OS curve in the placebo group, and the PFS and OS curves in the toripalimab group. The fitted parameters and fitted curves were shown in Supplementary Table 2 and Supplementary Figure 1, respectively.

### 2.3. Cost estimate

Because this study evaluated the cost-effectiveness of toripalimab plus chemotherapy based on the standpoint of the Chinese healthcare system, only direct medical costs were considered, including drug costs, adverse event management costs, best supportive care costs, hospitalization costs, and follow-up costs. The price of the drug was the average price of each province and city in the country, which was obtained from the China Medical Information Network.^3^ The costs of adverse event management, best supportive care, hospitalization and follow-up were based on the average price of medical services in Jiangsu province. Because grade 1 and 2 adverse reactions were not usually treated, the costs of adverse event management were calculated only for grade 3 and above. At the same time, to simplify the model, only adverse reactions with an incidence greater than 5% were considered in the first cycle. Hospitalization costs included bed costs, nursing costs, inpatient consultation costs, and chemical drug configuration costs, and assumed that hospitalization was 3 days each cycle. The follow-up costs included the costs of imaging examination (chest plain scan + enhanced CT) and laboratory examination (blood routine examination, complete set of biochemical tests, thyroid five items, urine routine examination, stool routine examination, electrocardiogram, coagulation five items, serum lung cancer five items, free β-chorionic gonadotropin measurement), and assumed that imaging examination was performed once in two cycles and laboratory examination was performed once in one cycle. See Table 1 for the cost information.

**Table 1:** Cost and utility parameters.

Variable	Baseline value	Low value	High value	Distribution	Source
Cost ($)					
Toripalimab per 240 mg	276.46	221.17	276.46	Gamma	Menet
Nabpaclitaxel per 30 mg	21.75	17.4	26.1	Gamma	Menet
Pemetrexed per 100 mg	70.5	56.4	84.6	Gamma	Menet
Carboplatin per 100 mg	8.43	6.74	10.11	Gamma	Menet
Cisplatin per 30 mg	3.29	2.63	3.95	Gamma	Menet
Docetaxel per 20 mg	41.37	33.1	49.65	Gamma	Menet
Anemia per event	5.53	4.42	6.64	Gamma	Local price
Thrombocytopenia per event	1,213.24	970.59	1,455.89	Gamma	Local price
Neutropenia per event	66.77	53.42	80.12	Gamma	Local price
Leukopenia per event	186.57	149.26	223.88	Gamma	Local price
Follow-up per cycle	240.19	192.15	288.23	Gamma	Local price
hospitalization per cycle	61.57	49.25	73.88	Gamma	Local price
Incidence of ARs					
Anemia in toripalimab group	0.299	0.239	0.359	Beta	(15)
Anemia in placebo group	0.359	0.287	0.431	Beta	(15)
Thrombocytopenia in toripalimab group	0.172	0.138	0.206	Beta	(15)
Thrombocytopenia in placebo group	0.179	0.143	0.215	Beta	(15)
Neutropenia in toripalimab group	0.555	0.444	0.666	Beta	(15)
Neutropenia in placebo group	0.538	0.43	0.646	Beta	(15)
Leukopenia in toripalimab group	0.357	0.286	0.428	Beta	(15)
Leukopenia in placebo group	0.417	0.334	0.5	Beta	(15)
Utility value					
PFS	0.804	0.536	0.84	Beta	(17)
PD	0.321	0.473	0.05	Beta	(17)
Disutility value					
Anemia	0.073	0.0,584	0.0,876	Beta	(17)
Thrombocytopenia	0.023	0.018	0.028	Beta	(18)
Neutropenia	0.2	0.16	0.24	Beta	(17)
Leukopenia	0.113	0.09	0.134	Beta	(18)
Others					
Discount rate	5%	0	8%	Beta	(16)

ARs, adverse reactions; PFS, progression-free disease; PD, progressive disease; placebo group, placebo + chemotherapy; toripalimab group, toripalimab + chemotherapy.

In calculating the drug dose, this study assumed that the patient’s body surface area (BSA) was 1.72m^2^ and creatinine clearance (CCR) was 70 ml/min (19, 20).

### 2.4. Utility estimate

Since the CHOICE-01 trial did not report the quality of life information of patients, the utility value in this study was based on the health utility value of Chinese NSCLC population in an international study, which was 0.804 for PFS and 0.321 for PD (17). In addition, the disutility values of adverse reactions were also derived from the published studies (Table 1).

### 2.5. Sensitivity analysis

One-way sensitivity analysis and probabilistic sensitivity analysis (PSA) were performed to explore the influence of parameter uncertainty on the model. One-way sensitivity analysis was conducted to evaluate the impact of changes in a single parameter on the results, while PSA was applied to simulate the impact of changes in multiple parameters at the same time. This study intended to set the variation range of important parameters in the model, such as relevant cost value, health utility value, incidence of adverse reactions and discount rate, so as to conduct one-way sensitivity analysis, and the results were presented in the tornado diagram. Since toripalimab is unlikely to increase in price, the base value was set as the high value and the low value is set as 80% of the base value. The range of health utility values for PFS and PD stages was obtained from the upper and lower limits of an international study (17). According to the recommendations of Chinese Pharmacoeconomic evaluation guidelines, the discount rate was set from 0 to 8% (16). The value ranges of other parameters were set to ±20% of the base value (Table 1). In the PSA, 1,000 Monte Carlo simulations were carried out according to the variable range as well as the parameter distribution and the results were presented as cost-effectiveness scatter plot and cost-effectiveness acceptability curve (CEA). In this study, the cost data followed the Gamma distribution, and the utility value and the incidence of adverse reactions followed the Beta distribution.

### 2.6. Subgroup analysis

The CHOICIE-01 trial showed a different survival benefit between squamous and non-squamous cancers. The median PFS in the toripalimab group was 8.1 months (placebo group: 5.6 months) for squamous cancer and 9.7 months (placebo group: 5.5 months) for non-squamous cancer (15). In addition, the cost of squamous and non-squamous NSCLC was also different. Therefore, a subgroup analysis 1 was performed to investigate the cost-effectiveness of toripalimab plus chemotherapy in squamous and non-squamous carcinomas.

The CHOICE-01 trial demonstrated that patients with high tumor mutational burden (TMB; 72.7% vs. 46.7%, median PFS: 13.1 vs. 5.5 months) had higher objective response rates (ORR) and median PFS than patients with low TMB (65.7% vs. 46.2%, median PFS: 8.3 vs. 6.5 months). Because the combination of toripalimab and chemotherapy provided different degrees of survival benefit for patients with high TMB and low TMB, a subgroup analysis 2 of the economic difference of toripalimab in combination with high and low TMB was conducted.

### 2.7. Scenario analysis

Standard parametric model is generally considered suitable for fitting and extrapolate survival curves when the situation is not complex, while the Royston/Parmar spline model, mixture cure model and non-mixture cure model are relatively flexible to apply (21). Accordingly, this study evaluated the effect of different model fitting and extrapolating survival curves on the results.

## 3. Results

### 3.1. Baseline results

The basic results are shown in Table 2. The placebo group obtained 2.164 LYs and 1.03 QALYs at a cost of $22,145, while the toripalimab group gained 3.561 LYs and 1.75 QALYs at the price of $43,197. Compared with the placebo group, the toripalimab group received 0.71 QALYs and costed $21,052 more. However, the additional cost was worth because ICER ($29,478/QALY) was lower than the WTP ($35,108/QALY).

**Table 2:** Base-case results.

Parameter	Cost ($)	Incremental cost ($)	LYs	QALYs	Incremental QALYs	ICER($/QALY)
Placebo group	22,145		2.164	1.03		
Toripalimab group	43,197	21,052	3.561	1.75	0.71	29,478

LYs, life years; QALYs, quality-adjusted life years; ICER, incremental cost-effectiveness ratio; placebo group, placebo + chemotherapy; toripalimab group, toripalimab + chemotherapy.

### 3.2. Sensitivity analysis results

**Figure 2:**
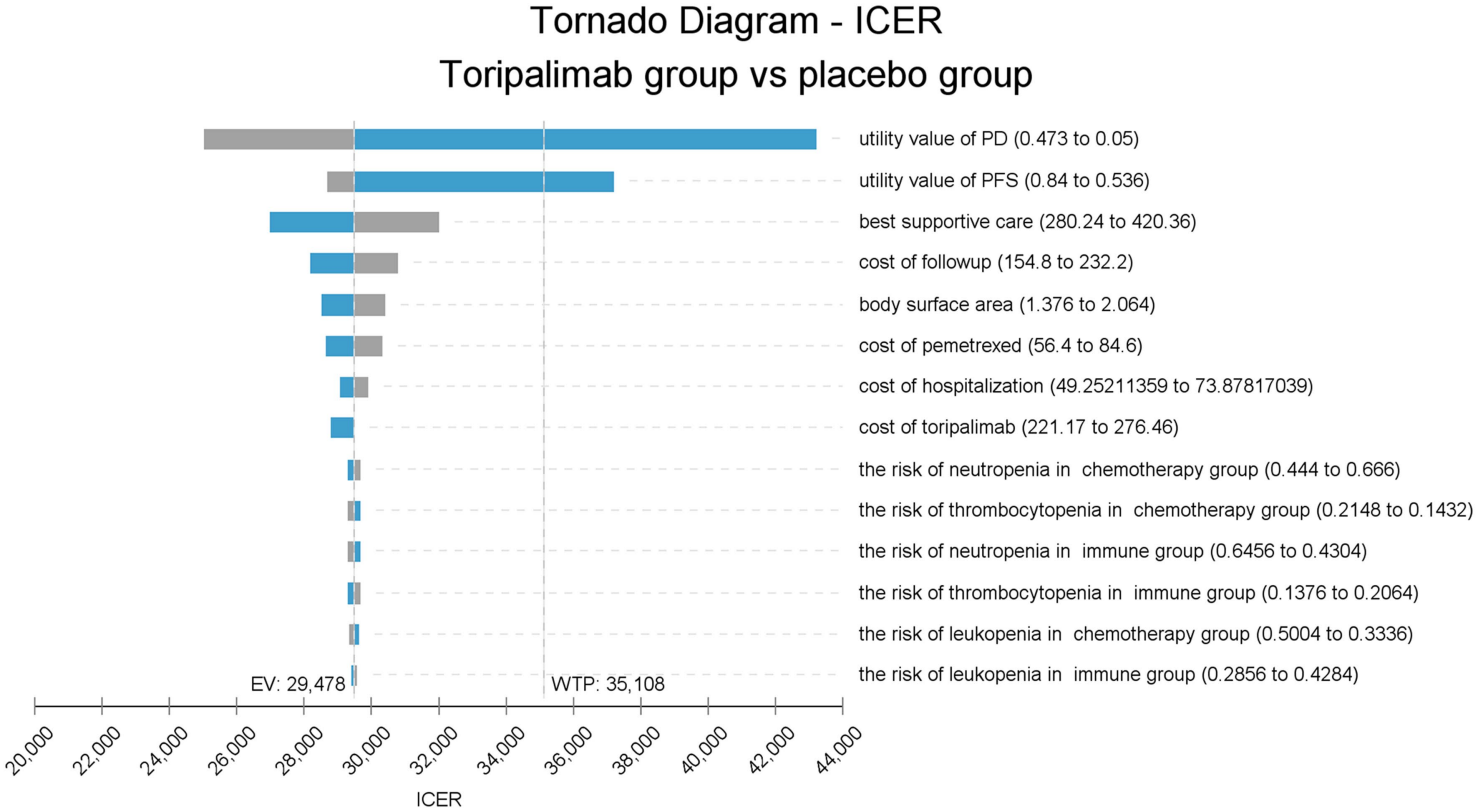
Tornado diagram of one-way sensitivity analysis. ICER, incremental cost-effectiveness ratio; placebo group, placebo + chemotherapy; toripalimab group, toripalimab + chemotherapy; PFS, progression-free survival; PD, progressive disease; EV, expected value; WTP, willingness-to-pay.

The tornado chart shows the parameters that have a great influence on ICER (Figure 2). From this figure, it can be seen that the utility values of PFS and PD stages have the greatest effect on the model. Lower utility values lead to a higher ICER, and even when two values approach their lower bound, ICER will be larger than the WTP, causing a reversal of results. Other parameters, such as cost of BSC, cost of follow-up, BSA, cost of hospitalization, and price of pemetrexed, had modest effects on the model but did not reverse the results.

**Figure 3:**
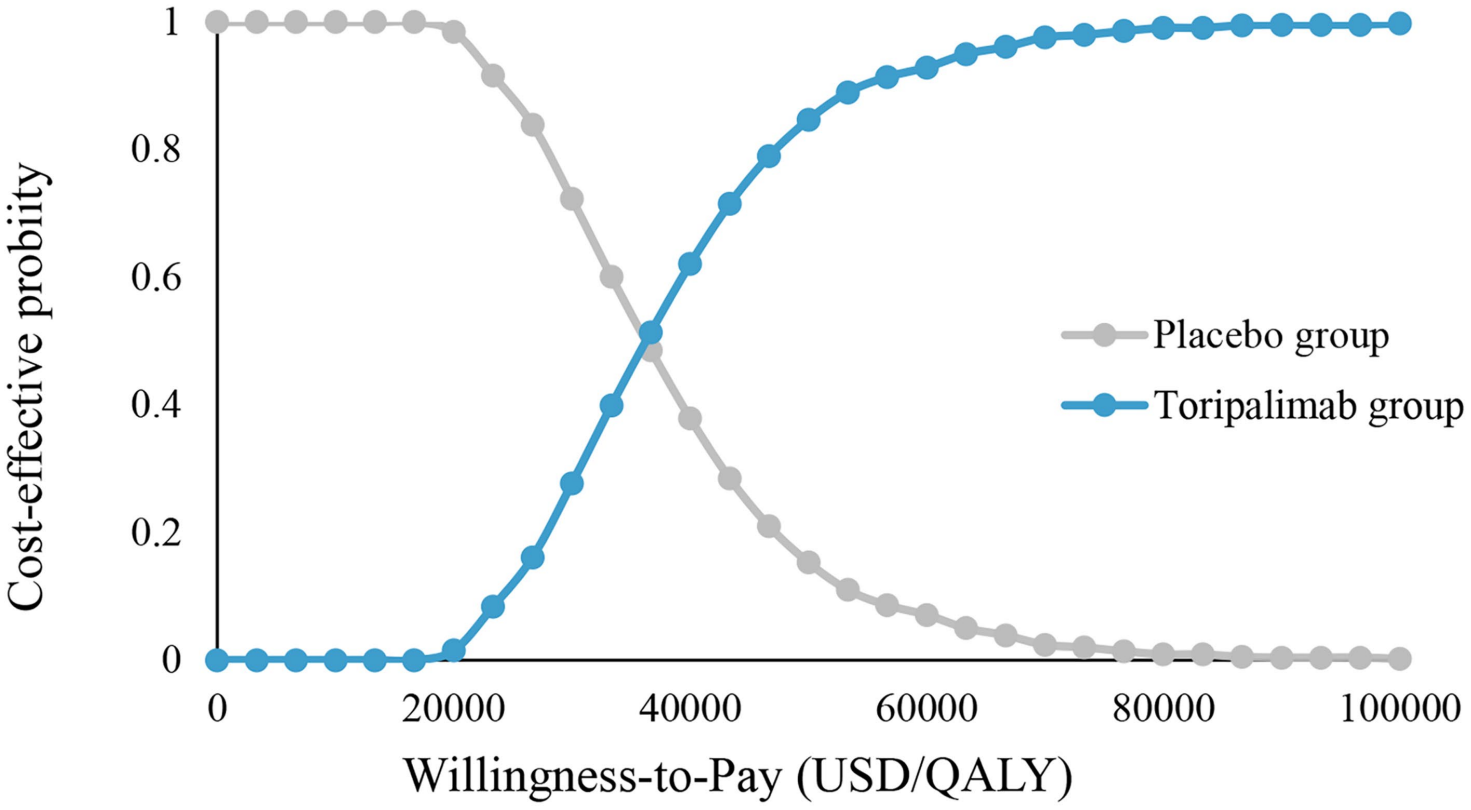
Cost-effectiveness acceptability curve. Placebo group, placebo + chemotherapy; toripalimab group, toripalimab + chemotherapy, QALY, quality-adjusted life year.

CEA (Figure 3) shows that toripalimab combined with chemotherapy starts to be cost-effective when WTP is $18,000/QALY. When the WTP is $36,000/QALY and $100,000/QALY, the economic probability of toripalimab coupled with chemotherapy was 50 and 100%, respectively.

The scatter plot (Supplementary Figure 2) represents the probability that toripalimab in combination with chemotherapy is cost-effective under the current WTP over 1,000 simulations. Out of 1,000 simulations, 463 points fell below the WTP line, indicating a 46.3% probability that toripalimab plus chemotherapy is cost-effective.

### 3.3. Subgroup analysis results

Subgroup analysis 1 showed that toripalimab in combination with chemotherapy provided 0.45 QALYs more than chemotherapy (0.95 QALYs vs. 1.4 QALYs) for patients with squamous NSCLC, with an incremental cost of $10,355 ($16,601 vs. $26,956), and the ICER was $23,258/QALY. In patients with non-squamous NSCLC, toripalimab plus chemotherapy generated 1.14QALYs more than chemotherapy (1.08 QALYs vs. 2.23 QALYs), and the additional cost was $35,585 ($23,255 vs. $58,841), with an ICER of $31,150/QALY. ICER was lower than the WTP in both squamous and non-squamous NSCLC, indicating that toripalimab coupled with chemotherapy has cost-effectiveness advantages in the treatment of both squamous and non-squamous NSCLC. See Table 3 for specific results.

**Table 3:** Subgroup and scenario analysis results.

Parameter	Cost ($)	Incremental cost ($)	LYs	QALYs	Incremental QALYs	ICER ($/QALY)
Subgroup analysis						
Squamous NSCLC						
Placebo group	16,601		2.005	0.95		
Toripalimab group	26,956	10,355	2.752	1.4	0.45	23,258
Non-squamous NSCLC						
Placebo group	23,255		2.167	1.08		
Toripalimab group	58,841	35,585	4.614	2.23	1.14	31,150
High TMB						
Placebo group	22,891		2.32	1.07		
Toripalimab group	46,583	23,692	3.59	2.12	1.05	22,544
Low TMB						
Placebo group	20,855		2.09	1.03		
Toripalimab group	43,893	23,039	3.71	1.76	0.73	31,731
Scenario analysis						
Royston/Parmar spline model						
Placebo group	22,189		2.164	1.03		
Toripalimab group	44,556	22,367	3.686	1.79	0.76	29,579
Mixture cure model						
Placebo group	30,990		3.13	1.34		
Toripalimab group	57,308	26,318	4.963	2.2	0.86	30,726
Non-mixture cure model						
Placebo group	29,449		2.943	1.29		
Toripalimab group	57,640	28,191	4.994	2.21	0.92	30,603

NSCLC, non-small cell lung cancer; TMB, tumor mutational burden; LYs, life years; QALYs, quality-adjusted life years; ICER, incremental cost-effectiveness ratio; placebo group, placebo + chemotherapy; toripalimab group, toripalimab + chemotherapy.

Subgroup analysis 2 (Table 3) showed that toripalimab plus chemotherapy yielded 1.05 QALYs more than chemotherapy alone in patients with high TMB (1.07 QALYs vs. 2.12 QALYs), and incremental cost was $23,692 ($22,891 vs. $46,583), with an ICER of $22,544/QALY. In patients with low TMB, toripalimab coupled with chemotherapy achieved 0.73 QALYs (1.03 QALYs vs. 1.76 QALYs) more than chemotherapy at a cost of $23,039 ($20,855 vs. $43,893), resulting in an ICER of $31,731/QALY. Therefore, regardless of TMB expression, adding toripalimab to chemotherapy was a cost-effective alternative to chemotherapy for advanced NSCLC.

### 3.4. Scenario analysis results

Scenario analysis (Table 3) showed that toripalimab in combination with chemotherapy has cost-effectiveness advantages in the treatment of advanced NSCLC, regardless of which model was applied to fit and extrapolate the survival curves. The results of the Royston/Parmar spline model most closely matched the standard parametric model, with 0.76 QALYs more gained with toripalimab group than with the placebo group (1.79 QALYs vs. 1.03 QALYs) and an ICER of $29,579/QALY. The results of the mixture cure model showed that the toripalimab group gained 0.86 QALYs more at an additional cost of $26,318, and the ICER was $30,726/QALY. The results of the non-mixture cure model showed that the toripalimab group and the placebo group gained 2.21 QALYs and1.29 QALYs, respectively, and the ICER was $30,603/QALY.

## 4. Discussion

Tumor immunotherapy is a rapidly developing new generation of tumor therapy after surgery, radiotherapy, chemotherapy, and other traditional therapeutic methods, which has a great prospect of clinical application (22, 23). Compared with traditional treatment methods such as surgery, radiotherapy, and chemotherapy, tumor immunotherapy has the advantages of strong specificity and small side effects. Immune checkpoint inhibitors (ICIs) are one of the main drugs of tumor immunotherapy in clinical practice (24). Toripalimab is the first approved domestic monoclonal antibody targeting PD-1 in China, which can block the PD-1 of T lymphocytes, block its binding to PD-L1 on the surface of tumor cells, relieve the immune suppression of T cells by tumor cells, and enable immune cells to regain their anti-tumor cell immune role and kill tumor cells (25, 26). It is worth mentioning that toripalimab is the only immunotherapy drug that can improve the 2-year survival rate of patients to more than 60% among the first-line immune combination therapies approved for non-squamous NSCLC patients in China, showing satisfactory survival benefits (15).

At present, more than 10 kinds of immunotherapy drugs have been approved for marketing in China. In the era of PD-1/PD-L1, how to choose an appropriate immunotherapy drug is not only considering the efficacy and safety of the drug but also the economy, especially for countries with limited medical resources. To the best of our knowledge, this study was the first to evaluate the economics of toripalimab combined with chemotherapy in the treatment of advanced NSCLC patients with negative driver genes based on the perspective of the Chinese healthcare system, and the results showed that toripalimab coupled with chemotherapy has cost-effectiveness advantages. Until toripalimab was approved for the treatment of advanced NSCLC, other domestic or foreign PD-(L)1 inhibitors approved for the treatment of advanced NSCLC were unlikely to be economical, such as camrelizumab, sugemalimab, and atezolizumab (27–29). The combination of toripalimab and chemotherapy was economical mainly because toripalimab was cheap and effective. (1) Toripalimab was the least expensive PD-1 inhibitor in its class at $276.46 per 240 mg (camrelizumab at $771.48 per 200 mg, sugemalimab at $1,788.42 per 600 mg, atezolizumab costs $4740.23 per 1,200 mg). (2) The CHOICE-01 trial showed that 62.6% of patients who received toripalimab plus chemotherapy lived longer than 2 years, compared with a 2-year OS rate of about 50% in similar product registry studies. Because toripalimab is not only effective but also affordable, it can be predicted that toripalimab had great application prospects in the future.

The CHOICE-01 trial showed that toripalimab plus chemotherapy was more effective in non-squamous NSCLC than in squamous NSCLC (15). The results of this study showed that although non-squamous cell carcinoma received more survival benefits, the economy was not as good as that of squamous cell carcinoma. This may be because non-squamous cell carcinoma used pemetrexed based chemotherapy, which was more expensive than other chemotherapy drugs. Fortunately, in the current WTP, toripalimab combined with chemotherapy has economic advantages in the treatment of both squamous and non-squamous cancer.

Some studies have found that patients with high TMB benefit more from immunotherapy (30–32). In 2020, the Food and Drug Administration (FDA) approved pembrolizumab for patients with solid tumors with high TMB, signaling the feasibility of TMB as a potential biomarker to screen people most likely to benefit from immunotherapy (33). The CHOICE-01 trial also showed that toripalimab combined with chemotherapy provided more survival benefit for patients with high TMB compared with patients with low TMB (15), and subgroup analysis in this study showed that the addition of toripalimab to first-line chemotherapy was economical for advanced NSCLC regardless of TMB, which suggested that using TMB to screen the population most likely to benefit from torpalimab therapy was not only effective but also worthwhile.

When evaluating drug economy, due to the short follow-up time of clinical trials, it is necessary to take appropriate methods to extrapolate long-term survival data based on K-M curves. The traditional practice is to use the standard parametric model for survival extrapolation, but the standard parametric model is only suitable for extrapolation when the situation is relatively simple (21). Because of the delayed effect of immunotherapy, standard parametric models often fail to accurately fit the survival curves (34, 35). Therefore, the National Institute for Health and Care Excellence developed some more flexible models to replace standard parametric models for fitting and extrapolating long-term survival from immunotherapy, such as flexible parametric (piecewise and cubic spline), mixture cure, parametric mixture, and landmark response models. To explore the differences in the results of fitting and extrapolating K-M curves from different models, standard parametric models, Royston/Parmar spline model, mixture cure model and non-mixture cure model were used in this study to fit and extrapolate survival curves. We found that toripalimab in combination with chemotherapy was economical for advanced NSCLC regardless of the model used. The differences in ICER values obtained by the four models were small, perhaps due to the immaturity of survival data. Regrettably, the lack of long-term survival data makes it impossible to judge which model best fits and extrapolates survival curves.

There are also some limitations in this study. First, there was inevitable uncertainty in extrapolating the long-term survival rate from the K-M curve. Second, because the CHOICE-01 trial did not report information on quality of life, we obtained health utility values from the published literature, which was unavoidable. Univariate sensitivity analysis showed that the utility values of PD and PFS stages had a great impact on ICER, suggesting that future clinical studies should not only pay attention to safety and efficacy, but also the quality of life of patients. Third, in order to simplify the model, we only included the cost of treatment for adverse events of grade 3 and above with an incidence greater than 5% in the calculation of the management cost of adverse events, which may result in a reduction in cost. However, one-way sensitivity analysis showed that even the most frequent adverse effects, such as neutropenia and leukopenia, had little effect on the model. Finally, adverse reactions between subgroups were not reported separately in the CHOICE-01 trial, so we did not account for the cost of managing adverse events and the resulting reduction in utility value in subgroup analyses. However, according to the tornado plot, it can be seen that neither the incidence of adverse reactions nor the treatment cost of adverse reactions nor the disutility value related to adverse reactions have almost no influence on the model. Therefore, it can be inferred that the results of the model would not change even if adverse reactions were not considered. Despite these limitations, our results may be useful for clinicians and patients when choosing appropriate therapeutic agents.

## 5. Conclusion

Based on the standpoint of healthcare system in China, when 3 times GDP *per capita* in 2021 was selected as the WTP, although adding toripalimab to the first-line chemotherapy for advanced NSCLC patients with negative driver genes cost more, it also obtained more survival benefits, and the disadvantage of cost could be compensated by survival advantages. Therefore, toripalimab combined with chemotherapy was cost-effective compared with chemotherapy.

## Data availability statement

The original contributions presented in the study are included in the article/Supplementary material, further inquiries can be directed to the corresponding author.

## Author contributions

HW and YL: conception and design. YZ: administrative support. HW and YX: provision of study materials or patients. YL and LL: collection and assembly of data. HW, YL, and LL: data analysis and interpretation. HW, YL, and YX: manuscript writing and final approval of the manuscript. All authors contributed to the article and approved the submitted version.

## Conflict of interest

The authors declare that the research was conducted in the absence of any commercial or financial relationships that could be construed as a potential conflict of interest.

## Publisher’s note

All claims expressed in this article are solely those of the authors and do not necessarily represent those of their affiliated organizations, or those of the publisher, the editors and the reviewers. Any product that may be evaluated in this article, or claim that may be made by its manufacturer, is not guaranteed or endorsed by the publisher.

## Footnotes

^1^ https://github.com/markummitchell/engauge-digitizer/releases

^2^ https://www.r-project.org

^3^ https://www.menet.com.cn
