# Supplementary material for: Economic evaluation of toripalimab combined with chemotherapy in the treatment of non-small cell lung cancer
Source: Front Public Health. 2023 Mar 24;11:1137255. doi: 10.3389/fpubh.2023.1137255 (PMC10080092; doi:10.3389/fpubh.2023.1137255)
Supplement: Supplementary file 1 [file Table_1.DOCX]

Supplementary Material

Economic evaluation of toripalimab combined with chemotherapy in the treatment of non-small cell lung cancer

Hao Wang ^1,2†^, Yunchun Long^1†^, Yuan Xu^2^, Li Liao^1^, Yujie Zhou^3*^

^1^ China Pharmaceutical University Nanjing Drum Tower Hospital, Nanjing, Jiangsu Province, China

^2^ Department of Pharmacy, Nanjing Drum Tower Hospital, Nanjing, Jiangsu Province, China

^3^ Department of Respiratory and Critical Care Medicine, Nanjing Drum Tower Hospital, Nanjing, Jiangsu Province, China

^†^ These authors have contributed equally to this work.

*** Correspondence:**

Yujie Zhou

yujie[zhoum@163.com](mailto:zhoum@163.com)

# Supplementary Figures and Tables

## Supplementary Figures

**Supplementary Figure 1.** Kaplan-Meier Curves Fitting and Extrapolation. A: PFS and OS curves of the toripalimab group, B: PFS and OS curves of the placebo group, PFS: Progression-free disease, OS: Overall survival, K-M: Kaplan-Meier


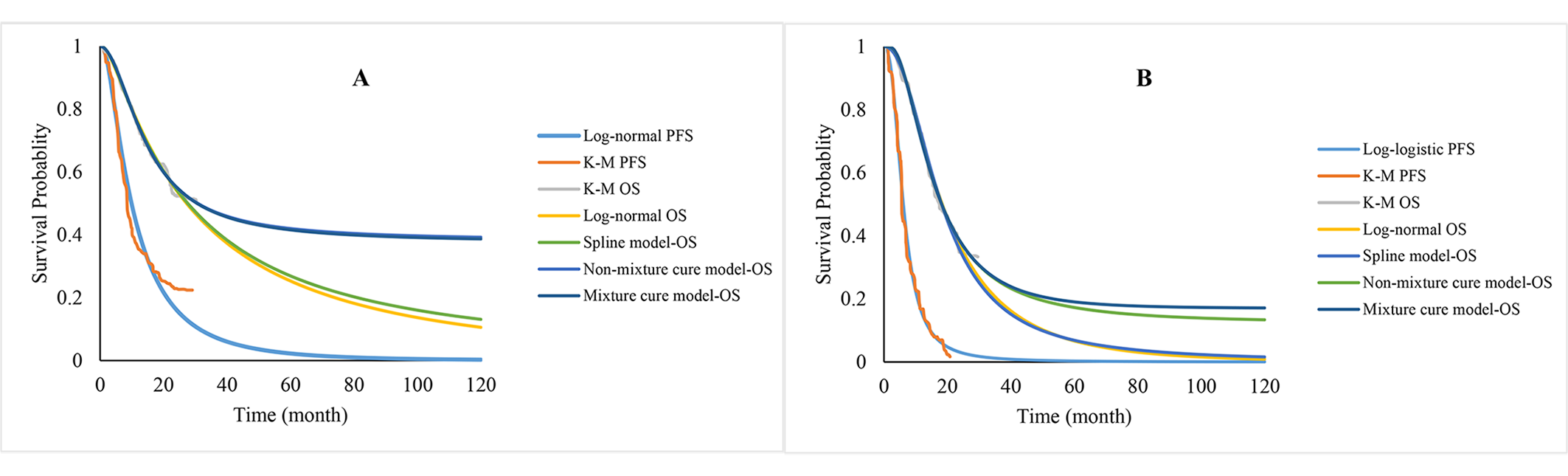


**Supplementary Figure. 2.** Scatter diagram. WTP: Willingness-to-pay, QALYs: Quality-adjusted life years.


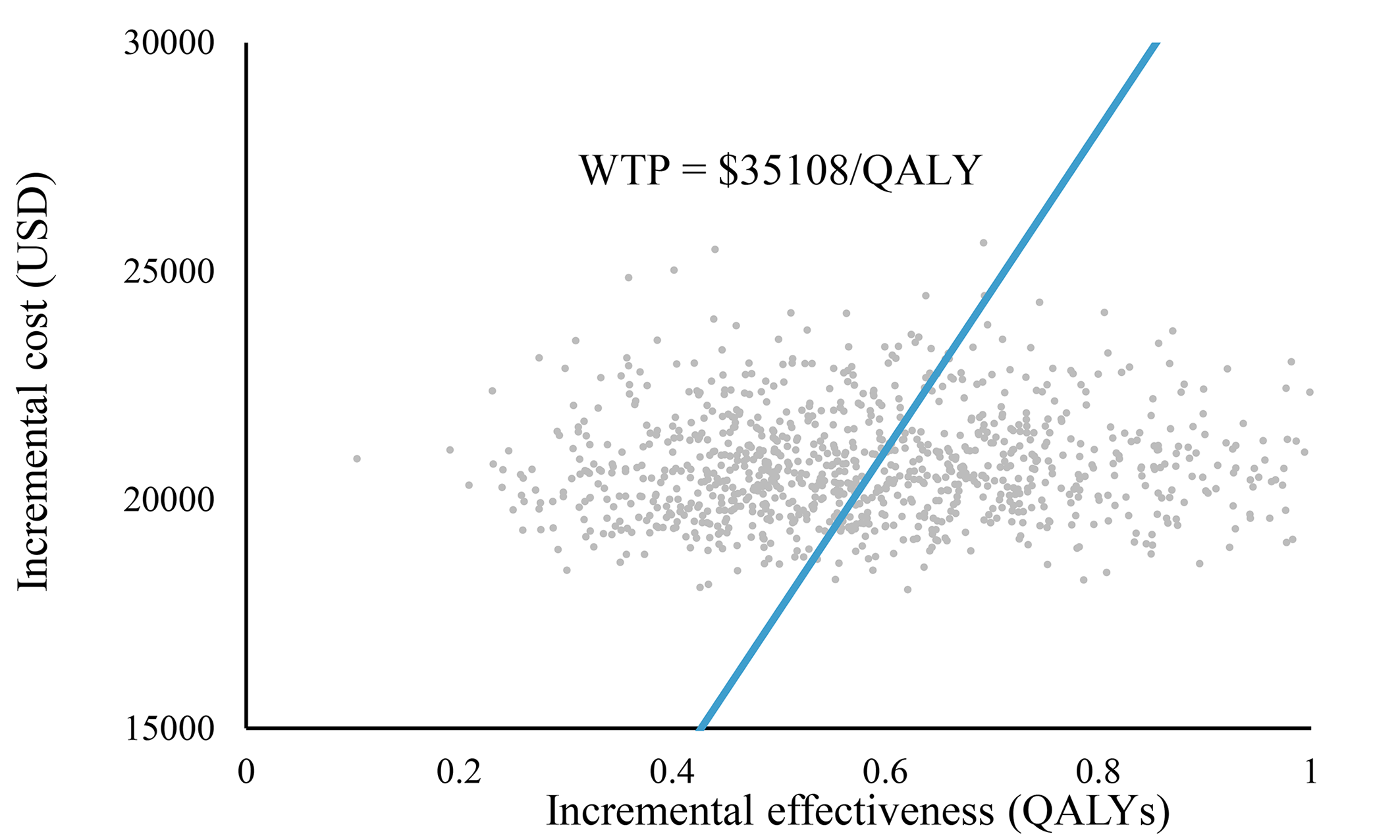


## Supplementary Tables

**Supplementary Table 1.** Function fitting results

|  | **Exponential** | **Weibull** | **Log-Logistic** | **Log-Normal** | **Gompertz** |
| --- | --- | --- | --- | --- | --- |
| **PFS of toripalimab group** |  |  |  |  |  |
| AIC | 1,423.563 | 1,404.107 | 1,369.3 | 1,365.695 | 1,424.546 |
| BIC | 1,427.296 | 1,411.574 | 1,376.766 | 1,373.162 | 1,432.012 |
| **PFS of placebo group** | |  |  |  |  |
| AIC | 814.708 | 775.004 | 762.104 | 762.493 | 796.815 |
| BIC | 817.758 | 781.104 | 768.204 | 768.593 | 802.915 |
| **OS of toripalimab group** |  |  |  |  |  |
| AIC | 1,104.786 | 1,096.497 | 1,092.257 | 1,090.35 | 1103.937 |
| BIC | 1,108.519 | 1,103.963 | 1,099.724 | 1,097.816 | 1111.403 |
| **OS of placebo group** | |  |  |  |  |
| AIC | 732.15 | 706.4 | 701.276 | 698.605 | 718.791 |
| BIC | 735.2 | 712.5 | 707.376 | 704.705 | 724.891 |

PFS: Progression-free survival, OS: Overall survival, AIC: Akaike Information Criterion, BIC: Bayesian Information Criterion, Placebo group: Placebo + chemotherapy, Toripalimab group: Toripalimab + chemotherapy

**Supplementary Table 2.** Best fit and the values of the parameters

| **Variable** | **Optimal distribution function** | **Baseline value** |
| --- | --- | --- |
| **PFS curve** |  |  |
| toripalimab group | log-normal | meanlog: 2.291,863，sdlog: 0.899,146 |
| placebo group | log-logistic l | λ: 6.10,374，γ: 2.51,876 |
| **OS curve** |  |  |
| toripalimab group | log-normal | meanlog: 3.30,488，sdlog: 1.18,733 |
| placebo group | log-normal | meanlog: 2.909,482，sdlog: 0.79,0421 |

PFS: Progression-free survival, OS: Overall survival, Placebo group: Placebo + chemotherapy, Toripalimab group: Toripalimab + chemotherapy, λ: Scale parameter, γ: Shape parameter
